# Kushen gel for the treatment of high-risk human papillomavirus infection: a systematic review and meta-analysis

**DOI:** 10.3389/fmed.2025.1707853

**Published:** 2025-11-06

**Authors:** Qingyun Li, Wenrui Huang, Xuelian Du, Ling Zhu

**Affiliations:** 1 Guangzhou University of Chinese Medicine, Guanzhou, Guangdong, China; 2 Shenzhen Traditional Chinese Medicine Hospital, Shenzhen, Guangdong, China; 3 The First Affiliated Hospital of Guangzhou University of Chinese Medicine, Guanzhou, Guangdong, China

**Keywords:** high-risk human papillomavirus, HPV clearance, Kushen gel, matrine and oxymatrine, systematic review and meta-analysis

## Abstract

**Background:**

Persistent high-risk human papillomavirus (HR-HPV) infection is the key driver of cervical cancer, yet current management strategies are often invasive or limited to watchful waiting. Kushen gel, derived from *Sophora flavescens*, has shown promise in promoting viral clearance, but clinical evidence remains inconsistent. This meta-analysis aimed to evaluate the efficacy and safety of Kushen gel in women with HR-HPV infection.

**Methods:**

We systematically searched PubMed, Embase, Cochrane Library, Web of Science, CNKI, Wanfang, VIP, and SinoMed databases from inception to September 2025 for randomized controlled trials (RCTs) evaluating Kushen gel in women with cervical HR-HPV infection. Eligible studies compared Kushen gel, either alone or in combination with interferon, loop electrosurgical excision (LEEP), or microwave therapy, against control interventions (follow-up or interferon). The primary outcome was HPV clearance, with secondary outcomes including viral load, recurrence, and adverse events. Subgroup analyses were prespecified according to treatment regimen, disease type, follow-up duration, and sample size. The risk of bias was assessed with the Cochrane tool, and certainty of evidence was evaluated using GRADE. Data were synthesized with random- or fixed-effects models depending on heterogeneity.

**Results:**

Nine RCTs involving 872 women were included. Pooled analysis showed that Kushen gel-based interventions significantly improved HPV clearance compared with controls (RR = 1.51, 95% CI 1.28–1.78), with consistent benefits across treatment regimens, disease types, follow-up durations, and study sizes. Kushen gel also significantly reduced HPV viral load (MD = −0.70, 95% CI -0.85 to −0.56), recurrence (OR = 0.21, 95% CI 0.09–0.52), and vaginal bleeding (OR = 0.29, 95% CI 0.14–0.60). Reported adverse events were mild and self-limiting, with no major between-group differences. Sensitivity analyses confirmed the robustness of the findings, whereas the GRADE certainty of evidence for HPV clearance was rated as low.

**Conclusion:**

Kushen gel significantly enhances HPV clearance and reduces recurrence with a favorable safety profile, supporting its potential as a non-invasive therapeutic option for women with HR-HPV infection.

**Systematic review registration:**

PROSPERO, CRD420251150231.

## 1 Introduction

Human papillomavirus (HPV) is the most prevalent sexually transmitted pathogen, and persistent infection has been firmly established as a principal cause of cervical cancer and its precursors (1). Among more than 200 HPV genotypes, approximately 14 are classified as high-risk (HR-HPV), with HPV-16 and HPV-18 accounting for the majority of cervical cancer cases (2). Although 80–90% of infections are transient and typically cleared by the host immune system within 2 years, persistent HR-HPV infection is a prerequisite for malignant transformation (1). The risk of persistence increases with age, particularly in postmenopausal women, in whom declining estrogen levels and immune senescence may impair viral clearance rates (3). These features underscore the pressing need for safe, effective, and non-invasive therapeutic options in this population.

In women diagnosed with low-grade lesions such as atypical squamous cells of undetermined significance (ASC-US), low-grade squamous intraepithelial lesions (LSILs), or cervical intraepithelial neoplasia grade 1 (CIN1), the prevailing management strategy is watchful waiting or active surveillance (4). While appropriate in many cases, this conservative approach may provoke considerable anxiety and require long-term follow-up. In contrast, high-grade lesions (CIN2/3, carcinoma *in situ*) are typically managed with ablative or excisional procedures such as loop electrosurgical excision (LEEP), cold-knife conization, or carbon dioxide (CO₂) laser conization (5). These techniques are effective in removing dysplastic tissue and preventing progression to invasive cancer; however, they are invasive and may compromise cervical integrity, increase the risk of preterm birth, and lead to psychological distress, particularly in younger or nulliparous women (6). Recent comparative studies suggest that CO_2_ laser conization may provide equivalent oncologic outcomes but with a lower rate of positive surgical margins compared with cold-knife procedures (7). Nevertheless, both approaches remain surgical in nature and do not directly target the underlying viral infection. Therefore, the development of safe, non-invasive local therapies capable of promoting HPV clearance and reversing early lesions—such as plant-derived formulations such as Kushen gel—addresses an important unmet clinical need.

*Sophora flavescens* Ait. (Kushen), a perennial herb belonging to the Fabaceae family, is widely distributed across East Asia and is used in traditional Chinese medicine for centuries to clear heat, dry dampness, and relieve itching (8). The dried roots are the main medicinal part and contain more than 200 identified compounds, primarily quinolizidine alkaloids such as matrine, oxymatrine, sophocarpine, and sophoridine (8–10). These alkaloids exhibit diverse pharmacological activities, including anti-inflammatory, antiviral, immunomodulatory, and antitumor effects, and may act synergistically to produce the overall therapeutic efficacy of *Sophora flavescens* preparations. To enhance mucosal retention and local drug delivery, the extract has been formulated into a carbomer-based vaginal gel—known as Kushen gel—which adheres to the cervical and vaginal mucosa, thus ensuring sustained release and high local bioavailability while minimizing systemic exposure (11, 12). Mechanistically, Kushen gel and its active alkaloids may inhibit HPV-related oncogenic processes by downregulating E6/E7 oncoprotein expression and restoring p53 and Rb tumor-suppressor pathways (13, 14), inducing cell cycle arrest and apoptosis in HPV-positive epithelial cells (15, 16). Concurrently, they modulate local immune responses by enhancing Th1-type cytokine production (e.g., IFN-*γ* and IL-12) (17) and activating Toll-like receptor 9 (TLR9) signaling, thereby facilitating viral recognition and clearance (18). These combined direct antiviral and immunomodulatory effects provide a biological rationale for the clinical application of Kushen gel in managing persistent high-risk HPV infection and its precancerous lesions.

These findings collectively support the potential of Kushen gel as a promising non-invasive therapeutic candidate for women with persistent HR-HPV infection and low-grade cervical lesions. Therefore, this systematic review and meta-analysis aims to synthesize and critically evaluate the available clinical evidence on the efficacy and safety of Kushen gel in women with cervical HR-HPV infection. By pooling data from multiple studies, we aim to provide more precise estimates of its therapeutic effects and to inform both clinical practice and future research directions.

## 2 Method

This systematic review and meta-analysis followed the PRISMA 2020 guidelines (Appendix 1) (19) and was prospectively registered in PROSPERO (CRD420251150231).

### 2.1 Data sources and search strategy

A comprehensive search was performed in PubMed, Web of Science, EMBASE, the Cochrane Library, China National Knowledge Infrastructure (CNKI), Wanfang Data, Weipu Data, and the Chinese Biomedical Literature Service System (SinoMed) databases from inception to September 2025 to identify randomized controlled trials (RCTs) evaluating Kushen gel for the treatment of cervical high-risk human papillomavirus (HR-HPV) infection. The search combined Medical Subject Headings (MeSH) and free-text terms using Boolean operators. Typical search logic included combinations such as (“human papillomavirus” OR “HPV” OR “high-risk HPV”) AND (“cervical intraepithelial neoplasia” OR “cervical dysplasia”) AND (“Kushen” OR “*Sophora flavescens*” OR “matrine” OR “oxymatrine” OR “gel”) AND (“randomized controlled trial” OR “clinical trial”), with animal-only studies excluded using the NOT operator. The detailed PubMed search string is provided in Appendix 2 as an example, with similar Boolean logic applied to other databases. Additional sources included reference lists of eligible studies, relevant clinical guidelines, conference proceedings, and trial registries.

### 2.2 Inclusion and exclusion criteria

Eligible studies were RCTs involving women with a confirmed diagnosis of cervical HR-HPV infection (20). Participants could be either married or unmarried, provided they had a history of sexual activity. Women with concurrent cervical intraepithelial neoplasia (CIN1, CIN2, or CIN3) were also eligible. Interventions included Kushen gel alone or in combination with recombinant human interferon-α2b (rhIFN-α2b), microwave therapy, or loop electrosurgical excision procedure (LEEP). Control groups consisted of interferon monotherapy, microwave or LEEP alone, or follow-up without medication. Eligible studies were those comparing Kushen gel-based regimens with these corresponding controls. Primary outcomes included HR-HPV clearance rate, HPV-DNA viral load, recurrence rate, vaginal bleeding, and other adverse events. The exclusion criteria were duplicate publications (with only the most complete or recent version retained), studies lacking explicit eligibility criteria, and trials involving patients with histologically confirmed cervical cancer, pregnancy, or lactation. Non-RCT designs, including reviews, retrospective analyses, and case reports were also excluded.

### 2.3 Data extraction and quality assessment

Two reviewers independently screened eligible studies, extracted data, and assessed methodological quality using the Cochrane Risk of Bias Tool. Extracted information included study characteristics (first author, year of publication, sample size), participant demographics, diagnostic criteria, treatment protocols, control interventions, and follow-up duration. Outcomes of interest were HR-HPV clearance rate, changes in HPV-DNA viral load, incidence of postoperative complications, and recurrence rate. Any discrepancies or disagreements during study selection, data extraction, or quality assessment were discussed to reach consensus; unresolved issues were adjudicated by a third senior reviewer to ensure accuracy and consistency.

### 2.4 Certainty of evidence assessment

The certainty of the evidence for each outcome was independently assessed using the Grading of Recommendations Assessment, Development and Evaluation (GRADE) approach, as recommended for systematic reviews of randomized controlled trials. This evaluation considered five domains: risk of bias, inconsistency, indirectness, imprecision, and publication bias.

### 2.5 Statistical analysis

All analyses were performed using aggregate data extracted from the published articles, as individual participant data (IPD) were not available. A meta-analysis was conducted using RevMan 5.4. For dichotomous outcomes, HPV clearance was expressed as risk ratios (RRs) with 95% confidence intervals (CIs), whereas recurrence and adverse events were reported as odds ratios (ORs) with 95% CIs. For continuous outcomes such as HPV-DNA viral load, the mean difference (MD) was used when measurement units were consistent across studies; otherwise, the standardized mean difference (SMD) was applied. Heterogeneity among studies was assessed using the chi-squared test and I^2^ statistic. If the *p*-value was > 0.10 and I^2^ was < 50%, a fixed-effects model was used; otherwise, a random-effects model was applied when the p-value of was ≤ 0.10 or I^2^ was > 50%, indicating significant heterogeneity. Sensitivity analysis was conducted by excluding individual studies to assess the robustness of the pooled results. When data permitted, subgroup analyses were performed according to treatment regimen (Kushen gel monotherapy, combined with interferon, combined with LEEP, or combined with microwave), disease type (chronic cervicitis, CIN I, CIN II/III, or persistent HR-HPV infection), follow-up duration (≤ 3 months vs. 6 months), and sample size (small, medium, or large trials) to explore potential sources of heterogeneity. Publication bias was assessed using funnel plots.

The total effect size for each outcome was defined as the pooled estimate (RR, OR, or MD) derived from the meta-analysis. Because the analysis was based on published summary statistics rather than raw data, tests for normal distribution or multiple-comparison adjustments (e.g., Bonferroni correction) were not applicable. All *p*-values correspond to pooled between-group comparisons across studies within the meta-analytic framework.

## 3 Results

### 3.1 Literature screening and included studies

**Figure 1:**
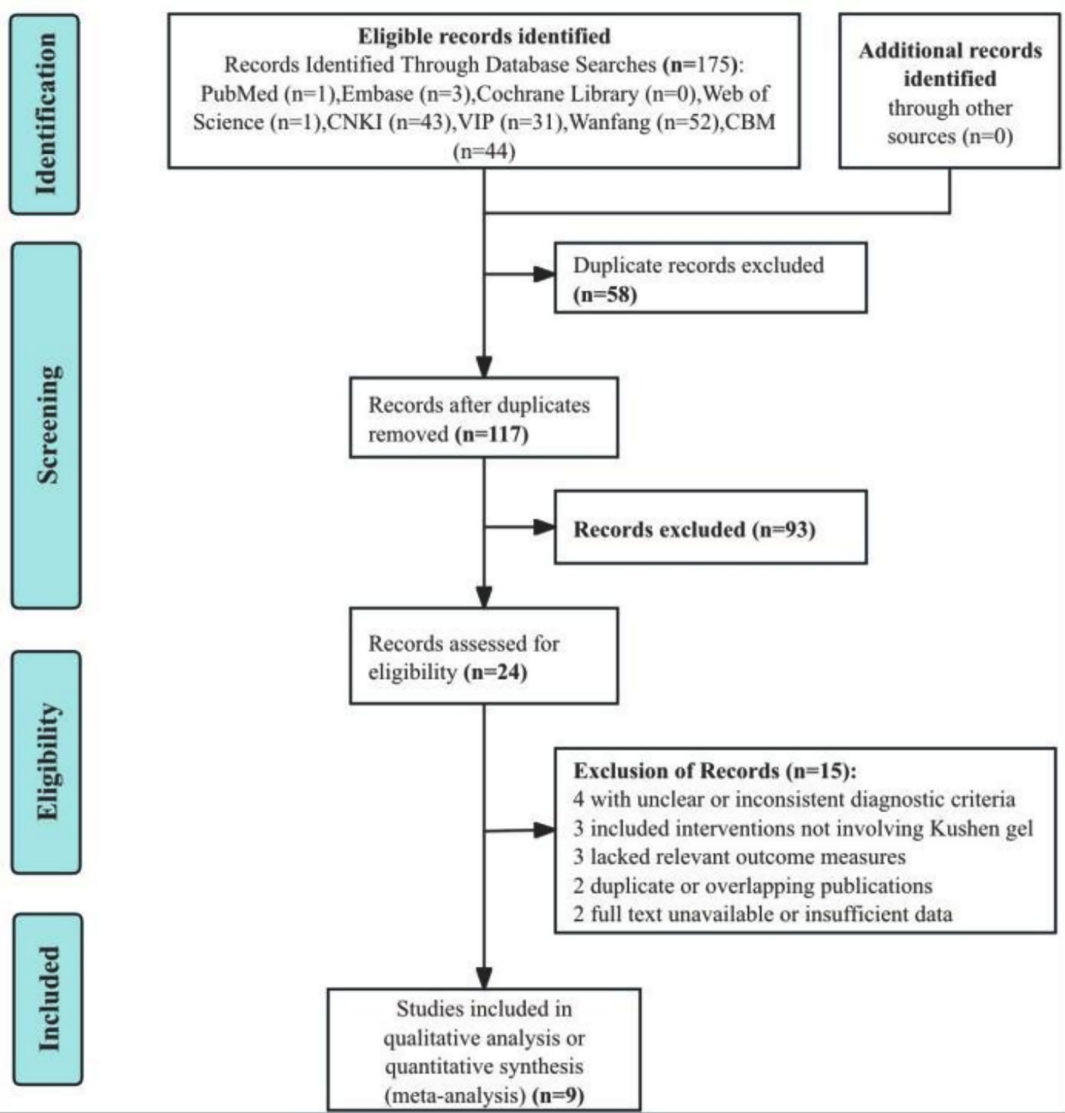
Flowchart shows study retrieval and inclusion.

A total of 175 records were initially retrieved from electronic databases. After removing 58 duplicates, 117 records remained for title and abstract screening. Of these, 93 studies were excluded for irrelevance to the topic or population of interest. The full texts of 24 articles were assessed for eligibility. Among them, 15 were excluded due to unclear or inconsistent diagnostic criteria (*n* = 4), interventions not involving Kushen gel (*n* = 3), lack of relevant outcome measures (*n* = 3), duplicate or overlapping publications (n = 2), or full text unavailable/insufficient data (*n* = 2). Ultimately, nine RCTs (21–29) were included in the qualitative and quantitative synthesis. The study selection process is depicted in the PRISMA flow diagram (Figure 1).

### 3.2 Characteristics of the included studies

A total of nine studies were included in this meta-analysis, all conducted in China between 2010 and 2023. The included populations encompassed women with chronic cervicitis, CIN, or persistent high-risk HPV infection. Sample sizes ranged from 30 to 76 participants per group, with follow-up durations varying from 3 weeks to 12 months. The mean age of participants across all studies ranged from approximately 31 to 40 years. Where reported, disease duration ranged from 13 months to 4 years, although some studies did not provide this information. One included trial (28) was a three-arm study (Kushen gel + rhIFN-α2b, Kushen gel, and rhIFN-α2b). For the purpose of the meta-analysis, it was treated as two separate comparisons: (1) Kushen gel + rhIFN-α2b vs. rhIFN-α2b and (2) Kushen gel vs. rhIFN-α2b (see Table 1).

**Table 1:** Baseline characteristics of the included studies.

Study	Country	Recruitment period	HPV	Age		Disease duration		Sample (T/C)	T	C	Follow up	Outcome
Pan et al., 2021 (21)	China	2018–12 to 2020–12	HPV (E6/E7 mRNA)	38.79 ± 4.22	38.86 ± 4.15	3.42 ± 1.09	3.26 ± 1.06	47/47	Kushen gel + rhIFN-α2b	rhIFN-α2b	6 m	①
Jie, 2022 (22)	China	2019–05 to 2021–08	High-risk HPV	31.45 ± 2.64	31.12 ± 2.13	1–4		36/36	Kushen gel + rhIFN-α2b	rhIFN-α2b	3 m	①③④⑤
Feng, 2016 (23)	China	2013–08 to 2014–05	Cervicitis with HR-HPV	33.5 ± 4.2	33.1 ± 4.7	3.0 ± 1.7	3.1 ± 1.3	52/52	Kushen gel + microwave	Microwave therapy	3 m	①④
Wan, 2021 (24)	China	2018–02 to 2021–03	Chronic cervicitis with HR-HPV	33.68 ± 3.12	33.80 ± 3.26	3.43 ± 1.02	3.46 ± 1.14	30/30	Kushen gel + rhIFN-α2b	rhIFN-α2b	3w	①
Zhou et al., 2019 (25)	China	2015–03 to 2017–07	CIN II/III with HR-HPV	35.79 ± 6.02	36.07 ± 5.92	NA		59/59	Kushen gel + LEEP	LEEP	6 m	①②③④⑤
Wang et al., 2025 (26)	China	2022–01 to 2023–04	Persistent HR-HPV	39.29 ± 3.94	39.84 ± 4.94	13 m		76/76	Kushen gel + LEEP	LEEP	3 m	①③⑤
Chen et al., 2016 (27)	China	2012–12 to 2014–09	CIN I with HR-HPV	37.5 ± 0.5	36.5 ± 0.5	NA		40/38	Kushen gel	No medication	3 m	①②
Zhao et al., 2016 (28)	China	2015–05 to 2016–05	CIN I with HR-HPV	NA		NA		40/40/40	Kushen gel + rhIFN-α2b/Kushen gel	rhIFN-α2b	3 m	①
Jin, 2014 (29)	China	2010–06 to 2013–06	Subclinical cervical HPV infection	34.3 ± 4.8	33.9 ± 4.3	17 m		37/37	Kushen gel + rhIFN-α2b	rhIFN-α2b	6 m	①⑤

T, treatment group; C, control group; not reported; m, months; w, weeks; HR-HPV, high-risk human papillomavirus; CIN, cervical intraepithelial neoplasia; LEEP, loop electrosurgical excision procedure; rhIFN-α2b, recombinant human interferon-α2b. Outcome measures: ① HPV clearance rate, ② HPV viral load, ③ recurrence rate, ④ vaginal bleeding, and ⑤ adverse events.

### 3.3 Risk of bias assessment

**Figure 2:**
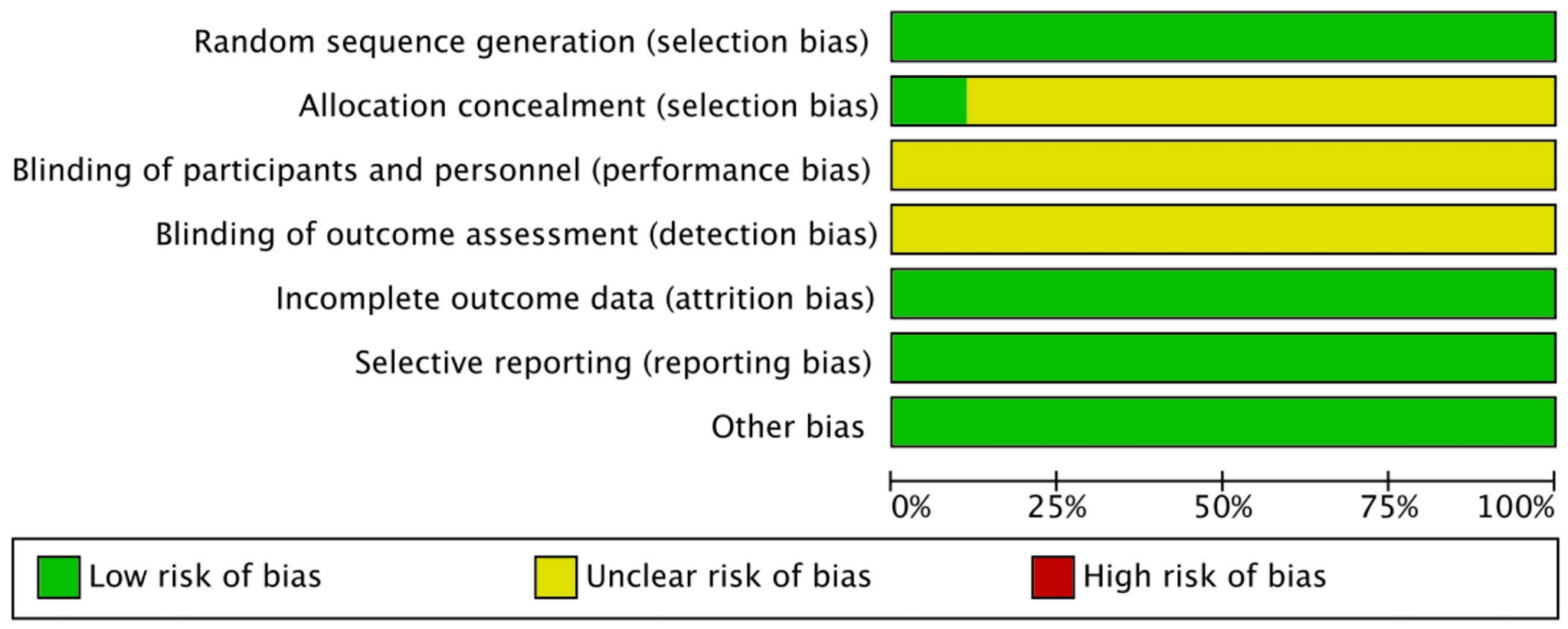
Risk of the bias graph.

**Figure 3:**
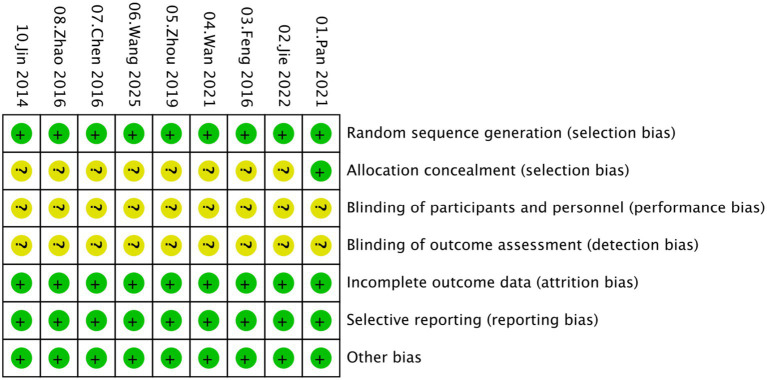
Risk of the bias summary.

Overall, the included studies showed a low risk of bias in domains related to incomplete outcome data and selective reporting, with all trials providing adequate follow-up and reporting protocols. Specifically, all studies were judged as low risk for both selective reporting and incomplete outcome data. For random sequence generation, most studies adequately described randomization and were rated as low risk. An unclear risk of bias was primarily concentrated in allocation concealment and blinding domains, as most studies did not provide sufficient details regarding these procedures (Figures 2, 3).

### 3.4 HPV clearance rate

**Figure 4:**
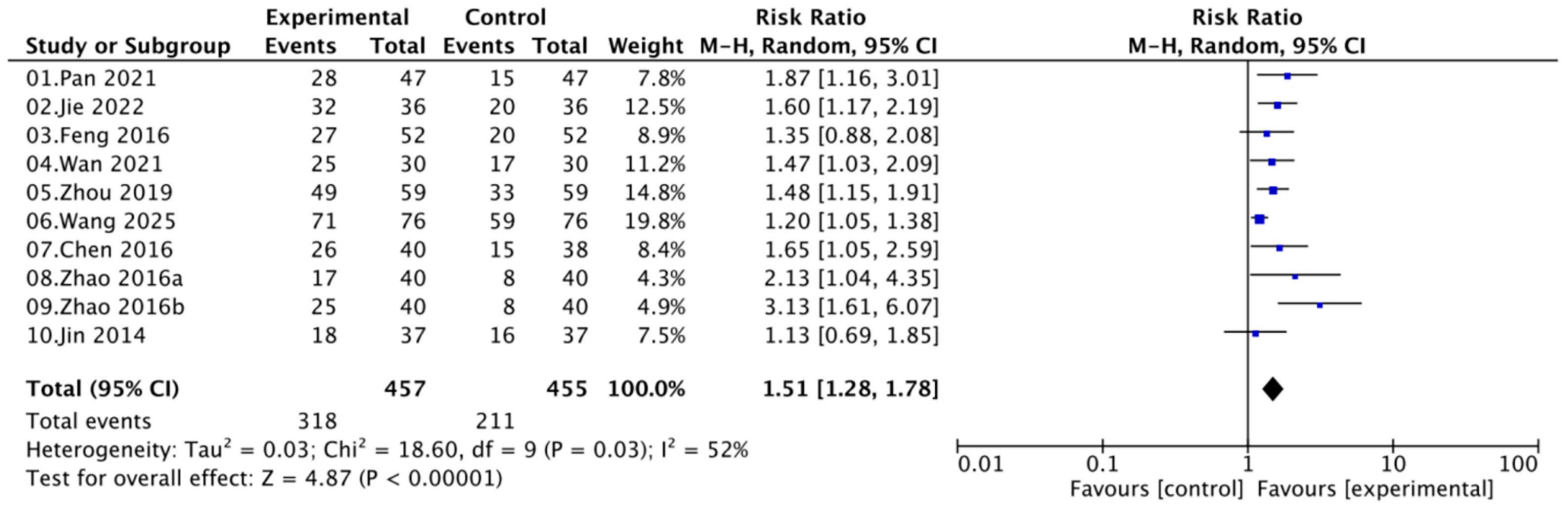
Forest plot of HPV clearance rate.

A total of nine studies involving 872 women (457 in the experimental groups and 415 in the control groups) reported on the HPV clearance rate. Moderate heterogeneity was observed (*I*^2^ = 52%, *p* = 0.03); therefore, a random-effects model was applied. Pooled analysis demonstrated that Kushen gel-based interventions significantly increased HPV clearance rate compared with controls (RR = 1.51, 95% CI [1.28–1.78]; *p* < 0.00001; Figure 4).

### 3.5 Subgroup analyses of HPV clearance

Subgroup analyses further explored the robustness of the findings (Appendix 3). When stratified by treatment regimen, Kushen gel monotherapy (RR = 1.77, 95% CI [1.21–2.60]), Kushen gel combined with interferon (RR = 1.63, 95% CI [1.27–2.10]), and Kushen gel combined with LEEP (RR = 1.30, 95% CI [1.05–1.62]) all showed significant advantages over controls, whereas the effect of Kushen gel combined with microwave was not statistically significant (RR = 1.35, 95% CI [0.88–2.08]) (Appendix 3, Figure 3.1).

When stratified by disease type, significant improvements in HPV clearance were observed in women with chronic cervicitis with HR-HPV (RR = 1.42, 95% CI [1.08–1.87]), CIN I (RR = 2.09, 95% CI [1.42–3.06]), CIN II/III (RR = 1.48, 95% CI [1.15–1.91]), and persistent HR-HPV without CIN (RR = 1.38, 95% CI [1.09–1.74]), indicating that Kushen gel provides consistent benefits across different pathological subgroups (Appendix 3, Figure 3.2).

Analysis by follow-up duration demonstrated that both ≤3 months (RR = 1.56, 95% CI [1.24–1.98]) and 6 months (RR = 1.48, 95% CI [1.19–1.82]) showed significant improvements in HPV clearance compared with controls, with no significant subgroup difference, suggesting stable efficacy over time (Appendix 3, Figure 3.3). Subgrouping by sample size revealed consistent benefits across small (≤40 per group, RR = 1.63, 95% CI [1.31–2.03]), medium (≈50–60 per group, RR = 1.51, 95% CI [1.24–1.85]), and large (≥70 per group, RR = 1.20, 95% CI [1.05–1.38]) trials, supporting the robustness of the pooled results (Appendix 3, Figure 3.4).

Collectively, these subgroup analyses confirmed that Kushen gel significantly enhances HPV clearance across different treatment strategies, disease severities, follow-up durations, and study sizes, with generally consistent effects and no evidence of effect modification.

### 3.6 HPV viral load

**Figure 5:**
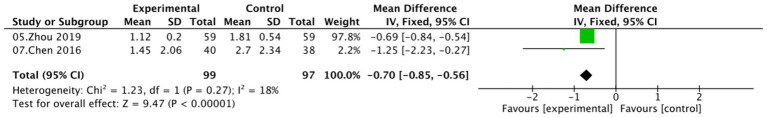
Forest plot of HPV viral load.

Analysis of HPV viral load was based on two studies (25, 27), including a total of 196 participants. Pooled analysis demonstrated a significant reduction in viral load in the Kushen gel group compared with controls (MD = −0.70, 95% CI [−0.85, −0.56]; *p* < 0.00001), with minimal heterogeneity across studies (*I*^2^ = 18%) (Figure 5). These findings suggest that Kushen gel therapy contributes to a more pronounced decrease in HPV viral replication.

### 3.7 Adverse outcomes

**Figure 6:**
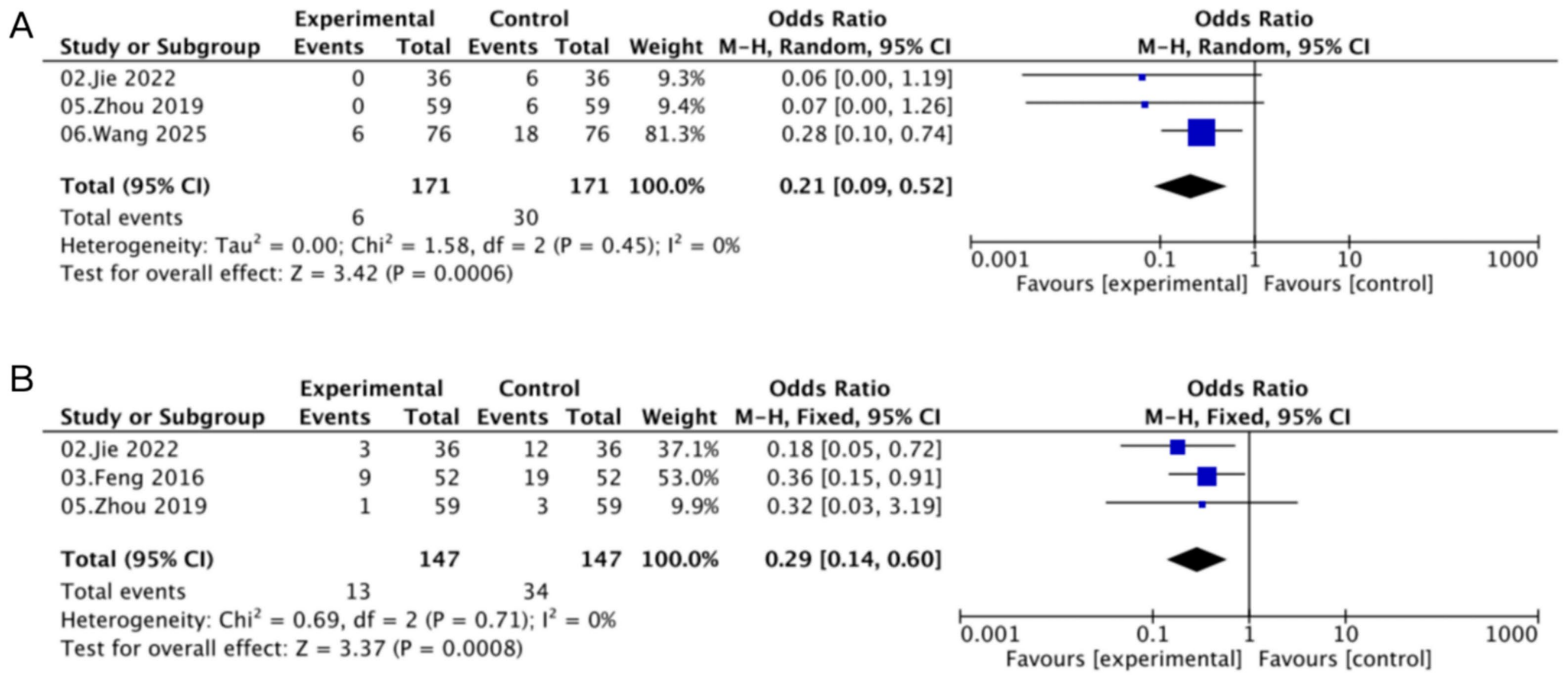
Forest plots of adverse outcomes: **(A)** recurrence rate and **(B)** vaginal bleeding.

Analysis of adverse outcomes demonstrated that Kushen gel significantly reduced the recurrence rate of HR-HPV infection compared with control interventions (OR = 0.21, 95% CI [0.09, 0.52]; *I*^2^ = 0%; Figure 6A). Similarly, the incidence of vaginal bleeding was markedly lower in the Kushen gel group (OR = 0.29, 95% CI [0.14, 0.60]; *I*^2^ = 0%; Figure 6B). These findings suggest that Kushen gel not only enhances viral clearance but also reduces the likelihood of recurrence and treatment-related adverse events.

In addition to recurrence and vaginal bleeding, several studies reported other adverse events qualitatively. Jie (22) documented mild symptoms in both groups, including pruritus, lower abdominal distension, burning sensation, dizziness, and nausea, with no notable between-group differences. Zhou et al. (25) observed comparable incidences of pruritus and vaginal edema, but the control group reported more cases of granulation tissue proliferation (4 vs. 0) and post-eschar bleeding (3 vs. 1). Wang et al. (26) recorded mild gastrointestinal symptoms, such as diarrhea, abdominal distension, and nausea, which were distributed across both groups without significant imbalance. Similarly, Jin (29) reported a lower frequency of mild vaginal pruritus in the Kushen gel group compared with the control group (1 vs. 3). Overall, these findings suggest that adverse events associated with Kushen gel were generally mild, self-limiting, and not significantly different from those observed in control groups.

### 3.8 Sensitivity analysis, publication bias, and evidence certainty

Sensitivity analysis was performed on the primary outcome of HPV clearance. Sequential exclusion of individual studies did not substantially alter the pooled effect size or direction of results, indicating that the findings were robust and not driven by any single study (Appendix 4). Publication bias was assessed using a funnel plot, which demonstrated a relatively symmetrical distribution of effect estimates, suggesting no obvious risk of publication bias (Appendix 5). The certainty of evidence for HPV clearance, as evaluated by the GRADE approach, was rated as low, primarily due to potential risk of bias and heterogeneity across included trials (Appendix 6).

## 4 Discussion

### 4.1 Primary findings

This meta-analysis of 9 RCTs involving 872 women demonstrated that Kushen gel-based interventions significantly improve HPV clearance compared with control treatments. Subgroup analyses confirmed the robustness of this effect across different therapeutic regimens, disease severities, follow-up durations, and study sizes, indicating broad potential clinical applicability. Beyond clearance, Kushen gel was also associated with reductions in HPV viral load, recurrence, and treatment-related adverse events, while reported side effects were generally mild and self-limiting. Collectively, these findings support Kushen gel as a promising adjunctive or alternative therapy for women with high-risk HPV infection.

### 4.2 Pharmacological mechanisms

In this meta-analysis, Kushen gel-based regimens significantly enhanced HPV clearance, reduced viral load, and lowered recurrence compared with control interventions, while adverse events were infrequent and mild. These clinical benefits are biologically plausible given the established pharmacological actions of its core alkaloids, matrine and oxymatrine. Unlike conventional single-target therapies, these compounds exert multitargeted effects that mirror the multifactorial pathogenesis of persistent HR-HPV infection. Experimental data indicate that matrine inhibits Akt/mTOR signaling, induces G2/M arrest, and promotes apoptosis through Bax upregulation and Bcl-2 suppression, thereby directly reducing the proliferative capacity of infected and dysplastic cells (15, 16). In parallel, modulation of E-cadherin and MMP2 expression constrains cellular migration and invasion, providing a mechanistic rationale for the observed reduction in recurrence (15).

The observed reduction in HPV viral load provides additional quantitative support for these biological effects. HR-HPV viral load is a critical biomarker of infection severity and progression risk (30). In our analysis, treatment with Kushen gel resulted in a significant decrease in viral load compared with controls (MD = −0.70, 95% CI [−0.85, −0.56]; *p* < 0.00001), indicating that the gel exerts a measurable inhibitory effect on viral replication. This finding aligns with evidence from a broader meta-analysis reporting that heat-clearing and dampness-eliminating Chinese medicine formulations, including *Sophora flavescens* extracts, markedly reduced HPV-DNA levels vs. interferon monotherapy (MD = −5.16, 95% CI [−5.91, −4.41]) (31). Such reductions demonstrate that Kushen gel’s benefits extend beyond achieving qualitative HPV negativity, representing a true suppression of viral replication. Mechanistically, these effects may result from the downregulation of E6/E7 oncoproteins, the restoration of p53 and Rb pathways, and the enhancement of local immune responses through Th1 cytokine activation and TLR9 signaling. Notably, other topical agents such as carrageenan-based gels failed to produce comparable effects on HR-HPV viral load (32), underscoring the biological specificity of Kushen gel’s active components.

Moreover, the favorable safety profile reflected in our pooled analysis may be partly attributable to Kushen gel’s anti-inflammatory and immunoregulatory properties, which not only mitigate local tissue injury but also enhance mucosal antiviral defenses. Formulation strategies incorporating complementary herbs—such as *Curcuma zedoaria* or *Scutellaria baicalensis*—or immune response modifiers such as imiquimod have shown synergistic effects in preclinical and early clinical studies (33). Collectively, these findings strengthen the biological plausibility and clinical significance of our results, supporting Kushen gel as a promising non-invasive therapeutic option for women with persistent HR-HPV infection.

### 4.3 Genotype-related variability in therapeutic response

While the above findings underscore Kushen gel’s broad antiviral and immunomodulatory potential, it remains unclear whether its therapeutic effects are consistent across different HPV genotypes. This question is clinically meaningful, as distinct HR-HPV types—particularly HPV16 and HPV18—differ in carcinogenic potential and biological behavior (30). Ideally, an effective intervention should demonstrate comparable efficacy across all major HR-HPV types, especially those with the highest oncogenic risk. In our meta-analysis, participants in the included studies were broadly described as women with “high-risk HPV infection,” “persistent HR-HPV positivity,” or “cervical intraepithelial lesions associated with HR-HPV infection,” including cohorts such as chronic cervicitis or CIN I–III patients with HR-HPV positivity or E6/E7 mRNA detection. However, none of the included trials specified individual HPV genotypes (e.g., HPV16, HPV18, and HPV52), and none reported genotype-stratified outcomes. Consequently, a subgroup analysis by HPV genotype could not be performed. Based on the available evidence, currently, no evidence indicates that the Kushen gel exerts differential effects among specific HPV genotypes. This constitutes an important evidence gap, highlighting the need for future trials to include genotype identification and stratified analyses to determine whether particular HPV types respond more favorably to treatment.

### 4.4 Heterogeneity and subgroup interpretation

Although the pooled analysis demonstrated a consistent overall benefit of Kushen gel-based therapy (RR = 1.51, 95% CI [1.28–1.78]), moderate heterogeneity was observed (*I*^2^ = 52%). To further explore potential sources of variability, subgroup analyses were conducted by treatment regimen, disease type, and follow-up duration. When stratified by treatment regimen, Kushen gel monotherapy (RR = 1.77, 95% CI [1.21–2.60]) and its combination with interferon (RR = 1.63, 95% CI [1.27–2.10]) demonstrated greater relative benefits compared with combination with LEEP (RR = 1.30, 95% CI [1.05–1.62]) or microwave therapy (RR = 1.35, 95% CI [0.88–2.08]). However, the test for subgroup differences showed no statistically significant variation across regimens (*I*^2^ = 0%, *p* = 0.40), indicating that treatment modality did not materially influence overall efficacy. By disease type, the greatest effect was observed in patients with CIN I (RR = 2.09, 95% CI [1.42–3.06]), followed by CIN II/III (RR = 1.48, 95% CI [1.15–1.91]) and persistent HR-HPV infection without CIN (RR = 1.38, 95% CI [1.09–1.74]). No significant subgroup difference was detected (*I*^2^ = 14.8%, *p* = 0.32), suggesting that Kushen gel provides consistent therapeutic benefits across various stages of cervical disease. Similarly, follow-up duration did not appear to affect outcomes: both ≤ 3 months (RR = 1.56, 95% CI [1.24–1.98]) and 6 months (RR = 1.48, 95% CI [1.19–1.82]) demonstrated stable efficacy, with no subgroup difference (*I*^2^ = 0%, *p* = 0.72). Overall, the moderate heterogeneity observed likely reflects methodological or population-level variation rather than true effect modification, reinforcing the robustness and generalizability of the pooled results.

### 4.5 Strengths and limitations

To our knowledge, this is the first meta-analysis to comprehensively evaluate the efficacy and safety of Kushen gel-based regimens for high-risk HPV infection. A major strength of our study lies in its systematic and rigorous methodology, including prespecified subgroup and sensitivity analyses across different disease severities, sample sizes, follow-up durations, and therapeutic strategies. We also applied GRADE to formally assess the certainty of evidence, thereby enhancing the transparency and reliability of our conclusions.

However, several limitations should be acknowledged. First, the overall certainty of evidence for the primary outcome (HPV clearance) was rated as low, primarily due to the risks of bias, small sample sizes, and moderate heterogeneity among studies. This indicates that the true effect may differ from the pooled estimate, and the observed benefits should be interpreted with caution. Second, all included trials were conducted in China, which may limit generalizability to other populations given potential ethnic and healthcare system differences. Third, reporting quality varied across studies, with incomplete descriptions of allocation concealment and blinding, introducing potential performance and detection bias. Finally, minor variations in outcome definitions (e.g., HPV clearance criteria) and follow-up durations across studies may partly explain the moderate heterogeneity observed, reflecting methodological rather than biological differences.

### 4.6 Clinical implications and future directions

This meta-analysis highlights Kushen gel as a promising adjunctive option for women with high-risk HPV infection. By significantly enhancing viral clearance, reducing recurrence, and maintaining a favorable safety profile, Kushen gel addresses key gaps in current therapeutic strategies. Unlike imiquimod, whose cervical application is limited by mucosal irritation, Kushen gel offers a more tolerable topical alternative. Emerging data also support its role as an adjuvant following excisional procedures such as LEEP, where it may help reduce postoperative viral persistence. Compared with physical adsorption gels such as DeflaGyn® or carrageenan, Kushen gel provides a mechanistically distinct, multitargeted approach that not only suppresses viral replication but also modulates epithelial regeneration and local immune responses, potentially reversing early dysplastic changes.

From a broader perspective, progression from HPV infection to cervical intraepithelial neoplasia and carcinoma is shaped by multiple interrelated factors—including viral genotype, host immune status, nutritional balance, and lifestyle exposures such as smoking or chronic inflammation (34). Recent evidence highlights that certain micronutrients (e.g., vitamins A, C, D, E, and trace elements such as zinc and selenium) may confer protective effects through immune modulation and oxidative stress reduction (34). Within such a multifactorial, risk-stratified framework, Kushen gel could be most valuable for patients at intermediate risk—particularly those with persistent HR-HPV infection or with mild to moderate lesions (CIN1–2) who are unwilling or unsuitable for excisional surgery.

However, the current evidence base remains limited by small, single-center studies and low-GRADE certainty for HPV clearance, emphasizing the need for cautious interpretation. Future research should prioritize multicenter, randomized controlled trials with standardized outcomes, longer follow-up to assess recurrence and progression, and cost-effectiveness analyses to support policy-level decision-making. Additionally, investigating genotype-specific responses, fertility outcomes after treatment, and potential synergies with nutritional or immunomodulatory interventions may further clarify the optimal role of Kushen gel in comprehensive cervical disease management.

## 5 Conclusion

This meta-analysis provides the first comprehensive synthesis of evidence indicating that Kushen gel-based therapies significantly enhance HPV clearance, reduce viral load and recurrence, and are generally well tolerated in women with high-risk HPV infection. These findings suggest that Kushen gel may serve as an effective adjunctive or alternative treatment alongside existing antiviral and surgical approaches. However, the certainty of evidence for HPV clearance was rated as low according to GRADE, primarily due to methodological limitations and study heterogeneity, underscoring the need for cautious interpretation. Moreover, none of the included trials reported genotype-specific outcomes, leaving it unclear whether the therapeutic efficacy varies among different HR-HPV genotypes. Future large-scale, multicenter RCTs with standardized outcome definitions, longer follow-up, and genotype-stratified analyses are warranted to confirm these benefits and better define the clinical role of Kushen gel in the global management of cervical HPV-related disease.

## Data Availability

The original contributions presented in the study are included in the article/Supplementary material; further inquiries can be directed to the corresponding author.
