# Supplementary material for: Kushen gel for the treatment of high-risk human papillomavirus infection: a systematic review and meta-analysis
Source: Front Med (Lausanne). 2025 Nov 6;12:1707853. doi: 10.3389/fmed.2025.1707853 (PMC12631416; doi:10.3389/fmed.2025.1707853)
Supplement: Supplementary file 1 [file Data_Sheet_1.docx]

**Appendix 1: Search strategy**

Search strategy of **PubMed**

#1 Uterine Cervical Neoplasms [mh] OR Uterine Cervical Dysplasia [mh] OR Papillomavirus Infections [mh]

#2 (cervical intraepithelial neoplasia) OR (CIN) OR (cervical lesions) OR (cervical dysplasia) OR (cervical cancer precursor) OR (HPV infection) OR (human papillomavirus) OR (high-risk HPV) OR (HR-HPV)

#3 (Sophora flavescens gel) OR (Kushen Gel) OR (Kushen ningjiao) OR (Sophora gel) OR (matrine hydrogel) OR (Sophora flavescens Aiton) OR (oxymatrine gel) OR (sophora flavescens alkaloid gel)

#4 (Randomized Controlled Trial [mh]) OR (controlled clinical trial [pt]) OR (randomized [tiab]) OR (placebo [tiab]) OR (randomly [tiab]) OR (groups [tiab]) OR (trial [tiab])

#5 animals [mh] NOT humans [mh]

#6 #1 OR #2

#7 #3 AND #4 AND #6 NOT #5

**Appendix 2. Subgroup analyses of HPV clearance rate**

Figure 2-1. Subgroup analysis by treatment modality (Kushen gel alone, Kushen gel + interferon, Kushen gel + microwave, Kushen gel + LEEP).


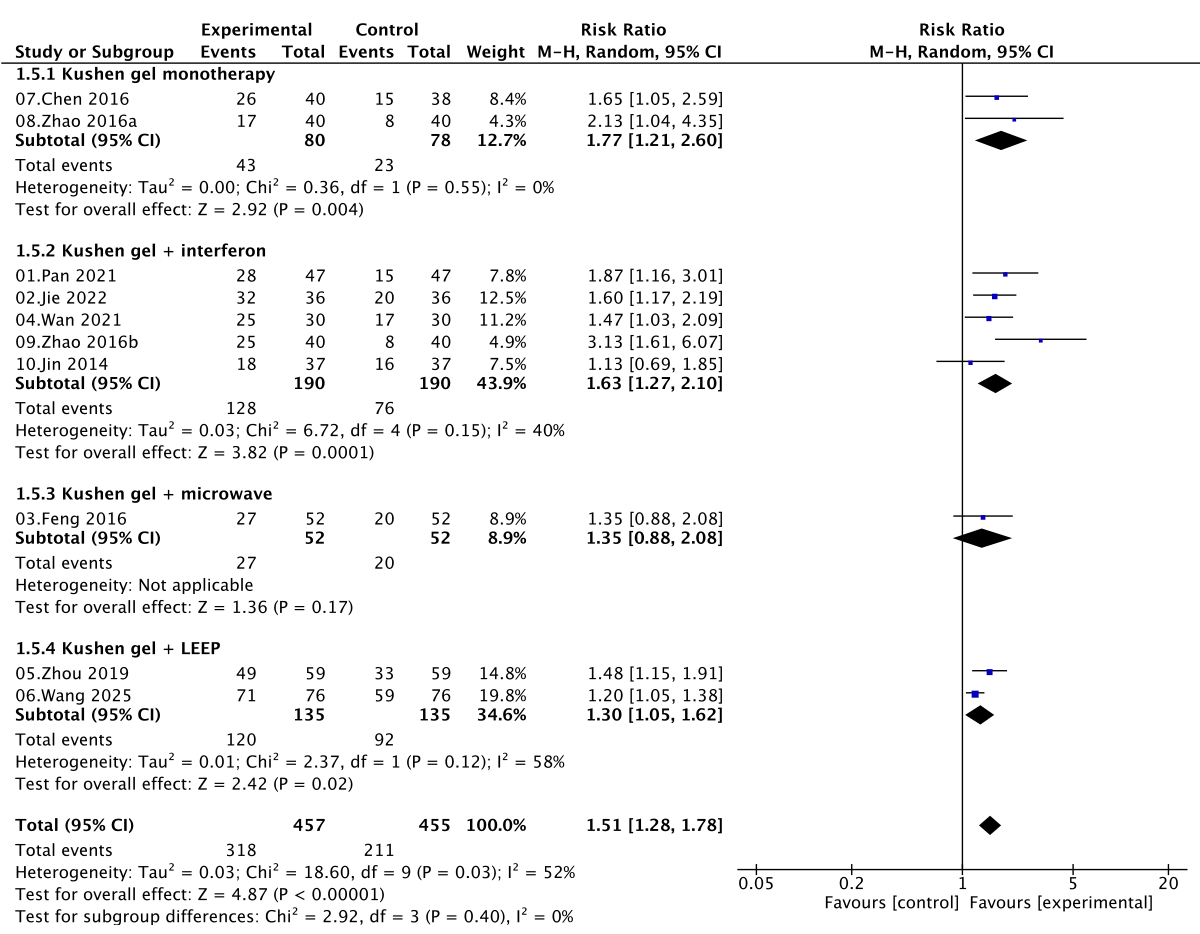


Figure 2-2. Subgroup analysis by pathological type (chronic cervicitis with HR-HPV, CIN I, CIN II/III, persistent HR-HPV without CIN).


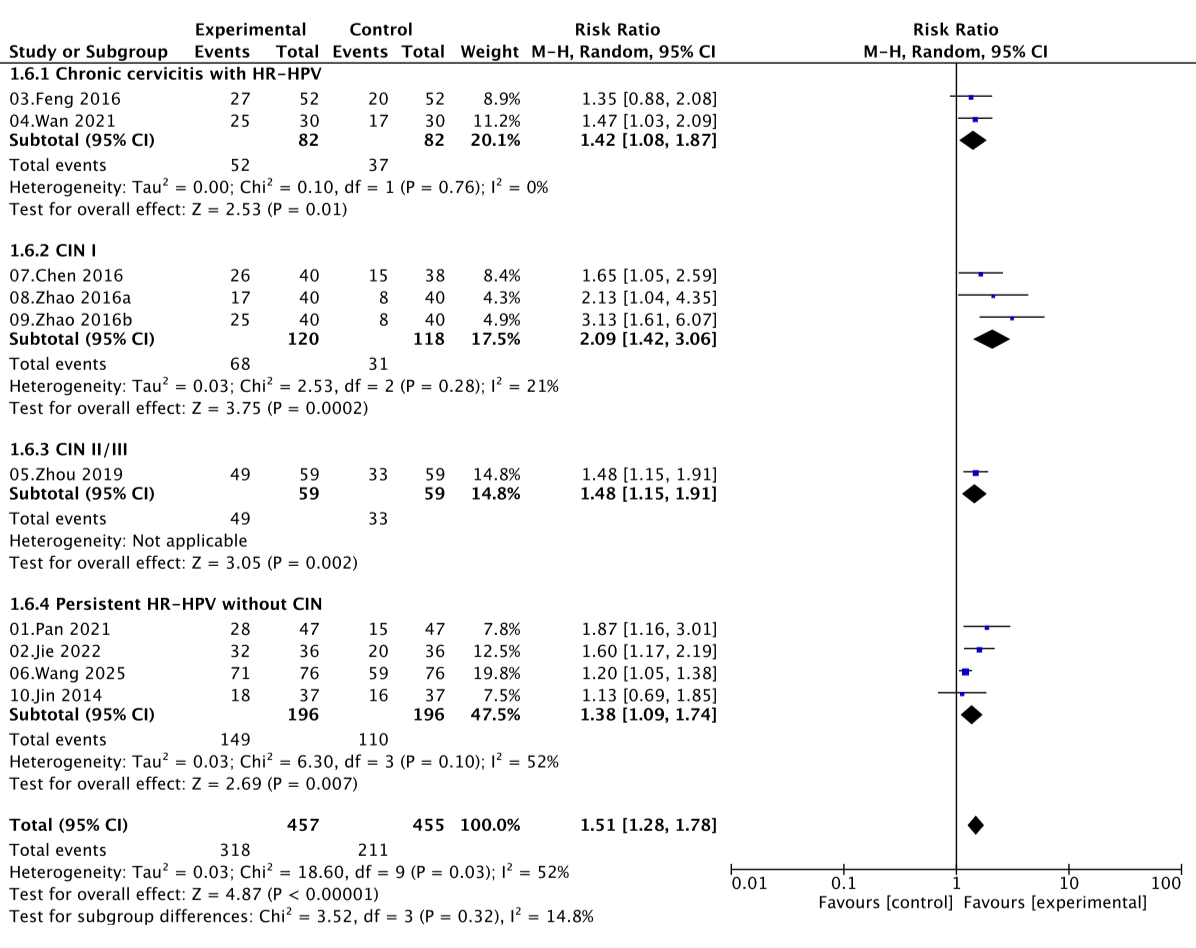


Figure 2-3. Subgroup analysis by follow-up duration (≤3 months vs. 6 months).


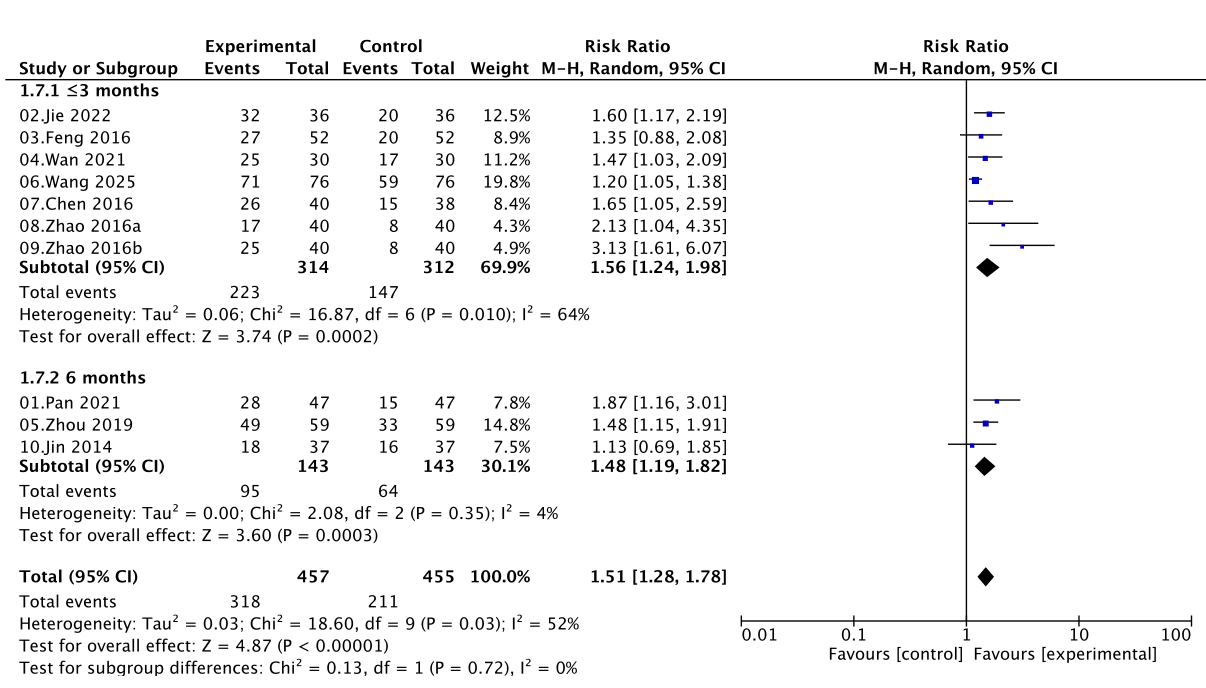


Figure 2-4. Subgroup analysis by sample size per group (≤40, 50–60, ≥70).


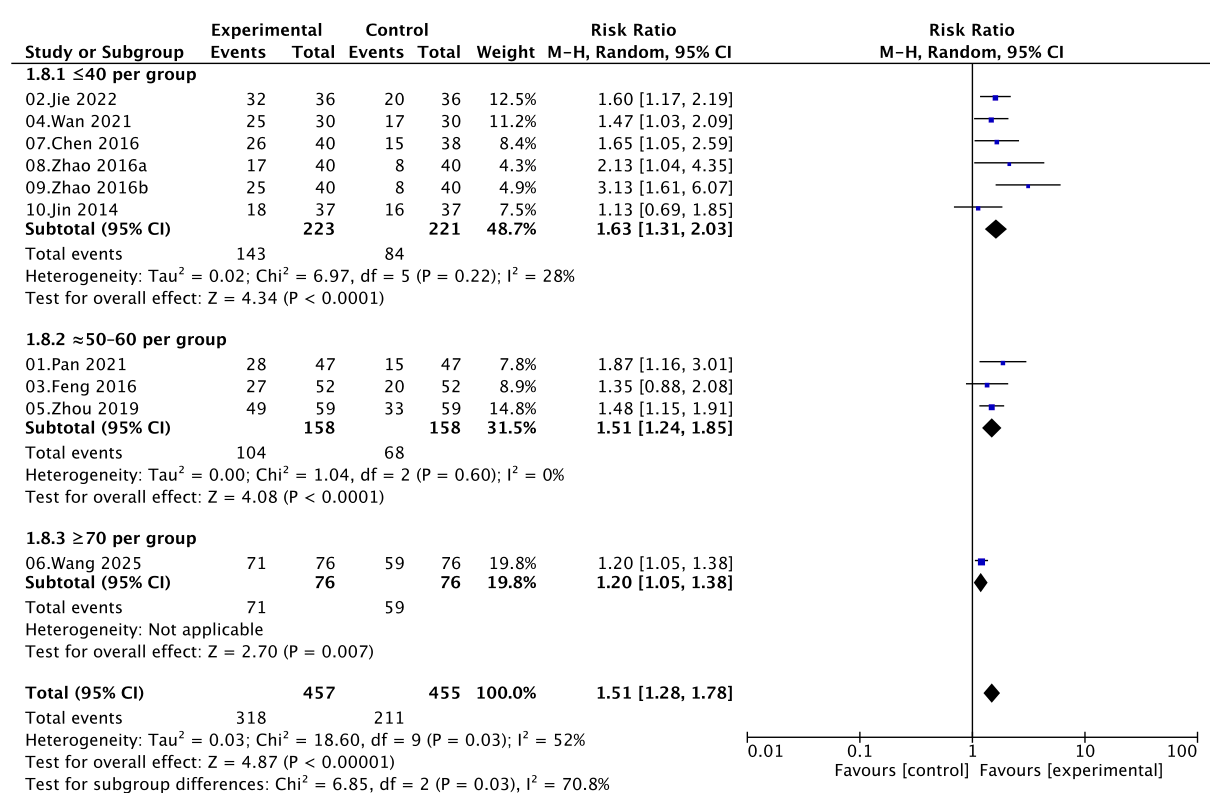


**Appendix 3: Sensitivity analysis of**


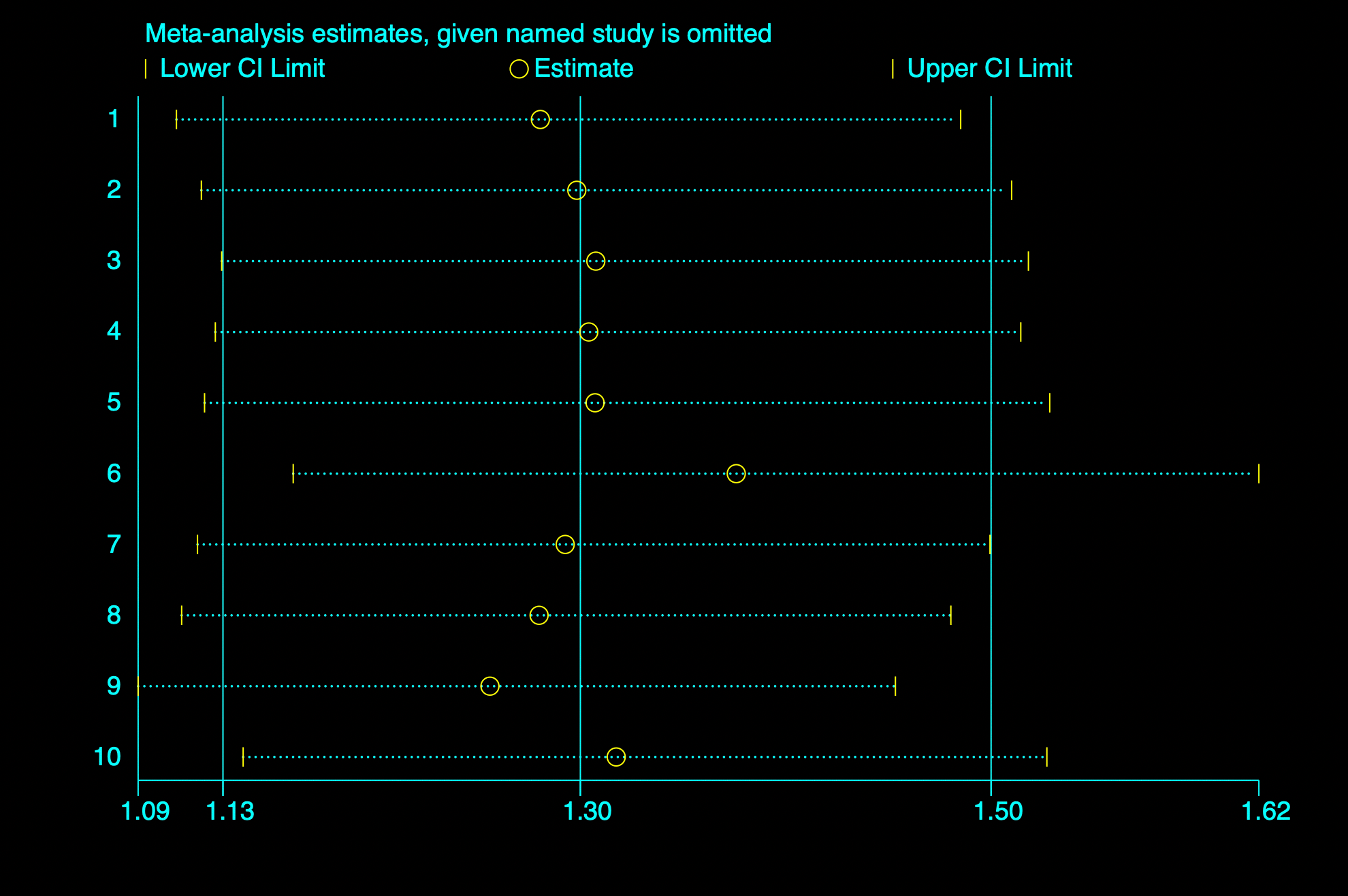


**Appendix 4: Funnel plot of**


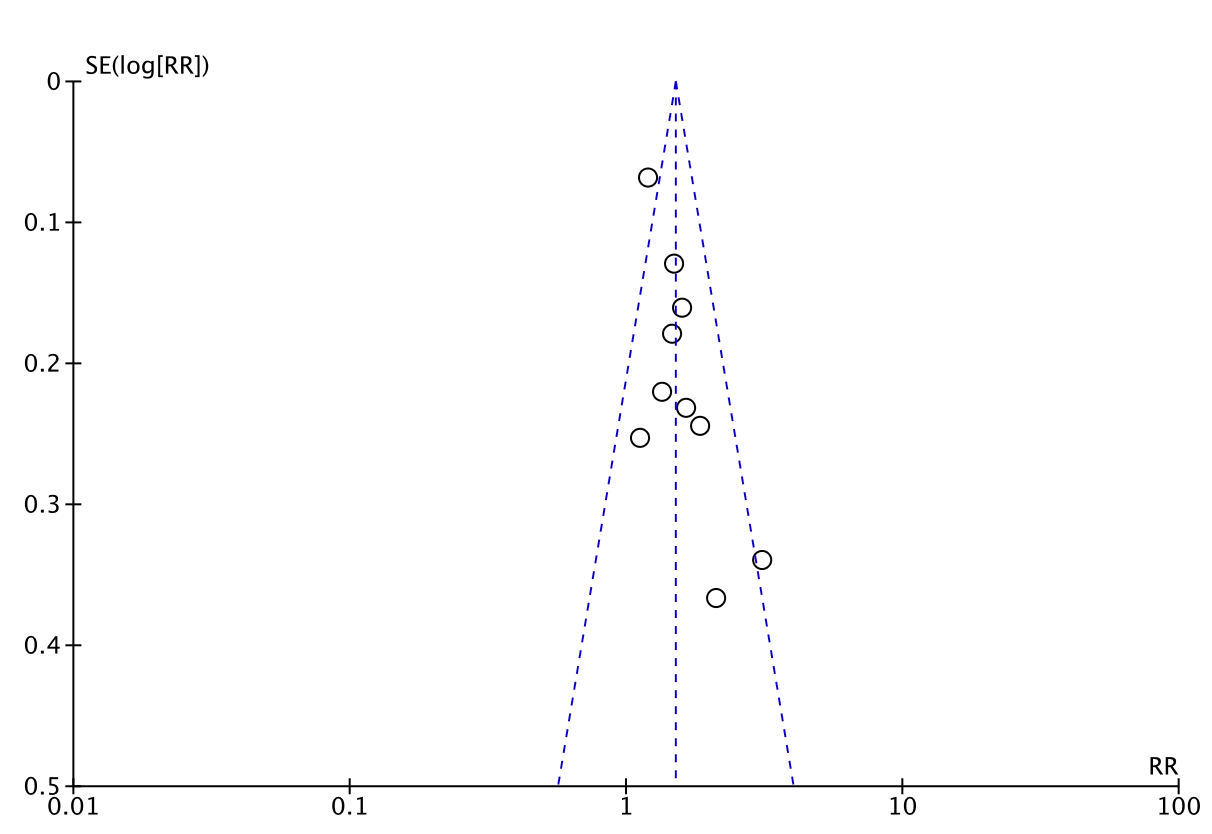


**Appendix 5：GRADE Evidence Profile for All Outcomes**

| **Certainty assessment** | | | | | | | **№ of patients** | | **Effect** | | **Certainty** | **Importance** |
| --- | --- | --- | --- | --- | --- | --- | --- | --- | --- | --- | --- | --- |
| **№ of studies** | **Study design** | **Risk of bias** | **Inconsistency** | **Indirectness** | **Imprecision** | **Other considerations** | **New Comparison** | **placebo** | **Relative (95% CI)** | **Absolute (95% CI)** |  |  |
| **HPV clearance rate** | | | | | | | | | | | | |
| 10 | randomised trials | serious | serious | not serious | not serious | none | 318/457 (69.6%) | 211/455 (46.4%) | **RR 1.50** (1.35 to 1.67) | **232 more per 1,000** (from 162 more to 311 more) | ⨁⨁◯◯ Low |  |
| **HPV viral load** | | | | | | | | | | | | |
| 2 | randomised trials | serious | serious | not serious | not serious | none | 99 | 97 | - | MD **0.7 lower** (0.85 lower to 0.56 lower) | ⨁⨁◯◯ Low |  |
| **Recurrence rate** | | | | | | | | | | | | |
| 3 | randomised trials | serious | serious | not serious | not serious | none | 6/171 (3.5%) | 30/171 (17.5%) | **OR 0.21** (0.09 to 0.52) | **133 fewer per 1,000** (from 157 fewer to 76 fewer) | ⨁⨁◯◯ Low |  |
| **阴道出血** | | | | | | | | | | | | |
| 3 | randomised trials | serious | serious | not serious | not serious | none | 13/147 (8.8%) | 34/147 (23.1%) | **OR 0.29** (0.14 to 0.60) | **151 fewer per 1,000** (from 191 fewer to 78 fewer) | ⨁⨁◯◯ Low |  |

**CI:** confidence interval; **MD:** mean difference; **OR:** odds ratio; **RR:** risk ratio
